# Rapid Screening of High-Yield Gellan Gum Mutants of *Sphingomonas paucimobilis* ATCC 31461 by Combining Atmospheric and Room Temperature Plasma Mutation with Near-Infrared Spectroscopy Monitoring

**DOI:** 10.3390/foods11244078

**Published:** 2022-12-16

**Authors:** Ling Sun, Yazhen Wang, Meixiang Yue, Xialiang Ding, Xiangyang Yu, Jing Ge, Wenjing Sun, Lixiao Song

**Affiliations:** 1 School of Food and Biological Engineering, Jiangsu University, Zhenjiang 212013, China; 2 Institute of Food Safety and Nutrition, Jiangsu Academy of Agricultural Sciences, Nanjing 210014, China; 3 Jiangsu Key Laboratory for Food Quality and Safety—State Key Laboratory Cultivation Base, Ministry of Science and Technology, Nanjing 210014, China

**Keywords:** gellan gum, fast screening, atmospheric and room temperature plasma (ARTP), Near Infrared Spectroscopy, *Sphingomonas paucimobilis*

## Abstract

In this study, an efficient mutagenesis and rapid screening method of high-yield gellan gum mutant by atmospheric and room temperature plasma (ARTP) treatment combined with Near-Infrared Spectroscopy (NIRS) was proposed. A NIRS model for the on-line detection of gellan gum yield was constructed by joint interval partial least squares (siPLS) regression on the basis of chemical determination and NIRS acquisition of gellan gum yield. Five genetically stable mutant strains were screened using the on-line NIRS detection of gellan gum yield in the fermentation from approximately 600 mutant strains induced by ARTP. Remarkably, compared with the original strain, the gellan gum yield of mutant strain 519 was 9.427 g/L (increased by 133.5%) under the optimal fermentation conditions, which was determined by single-factor and response surface optimization. Therefore, the method of ARTP mutation combined with the NIRS model can be used to screen high-yield mutant strains of gellan gum and other high-yield polysaccharide strains.

## 1. Introduction

Gellan gum, an extracellular polysaccharide with excellent physical and chemical properties, is produced by the aerobic fermentation of *Sphingomonas paucimobilis* (*S. paucimobilis*, formerly *Pseudomonas Elodea*) [1], in particular strain ATCC 31461 [2]. It is composed of four monosaccharide molecules: β-1,3-D-glucose, β-1,4-D-glucuronic acid, β-1,4-D-glucose, and α-1,4-L-rhamnose [3]. The natural gellan gum, directly produced by *S. paucimobilis*, is high-acyl gellan gum, which is soft and elastic. High-acyl gellan gum form low-acyl gellan gum after all or part of the acyl groups are removed in alkaline conditions, which is stronger, harder, more brittle, and stable in acidic conditions. Gellan gum has been widely used in food, medicine, chemical industry and other industries, and its product types are becoming more abundant [4,5,6]. However, compared with microbial exopolysaccharides such as xanthan gum, the price of gellan gum is higher, which greatly limits the wide application of gellan gum in the industry. The lower yield of gellan gum is the main reason for the higher prices.

Genetic modification of microorganisms is an important method to improve yield. Li et al. [7] combined UV irradiation and ethyl methanesulfonate (EMS) mutagenesis treatment to elevate gellan gum production of a double gene knockout mutant with unexpected production and obtained a mutant strain with production that was 132.8% higher than the double gene knockout strain and 14.4% higher than the wild-type strain ATCC 31461. Li et al. [8] proved that ampicillin, as a stressor and a mutagen, improved exopolysaccharides productivity and viscosity of *S. paucimobilis* ATCC 31461. In recent years, atmospheric and room temperature plasma (ARTP) mutation technology emerged as a new mutation breeding technology, which has been successfully applied in microorganisms [9,10,11,12,13]. Xu et al. [10] combined ARTP mutagenesis with 2,4-dinitrophenol (DNP) selection to develop lager yeast and obtained a series of mutant strains with higher NADH levels as well as improved flavor stability. Nyabako et al. [11] used ARTP mutation coupled with adaptive laboratory evolution (ALE) to improve the acid tolerance of *lactobacillus acidophilus* and obtained the mutant strain LAartp-ale2 with increased lactic acid stress tolerance. Cai et al. [12] reported the improved level of the tyrosine biosynthesis pathway in *Saccharomyces cerevisiae* through HTZ1 knockout and ARTP mutagenesis. Wang et al. [13] combined ARTP mutation with microtiter plate cultivation to screen lycopene-overproducing mutants of *Blakeslea trispora* and obtained a mutant (WY-239) showing a maximum lycopene concentration of 21.80 ± 1.58 mg/g. During the mutation process of ARTP, the produced high concentration of neutral active ions can penetrate the cell membrane, enter the nucleus, and directly act on chromosomes to cause gene damage, resulting in various mutations [9]. It has the advantages of simple operation, high mutation rate, and high genetic stability. Moreover, it is friendly to operators without using toxic and harmful substances in the operation process. ARTP mutagenesis of *S. paucimobilis* is expected to obtain a mutant with higher yield of gellan gum. However, it is a great workload to screen high-yield gellan gum mutants from various types of mutants induced by ARTP.

Near-Infrared Spectroscopy (NIRS) is a simple, rapid, and nondestructive analytical technique. NIRS can distinguish the absorption of frequency doubling and combining of different groups and then reflect it on the spectrogram. It has been widely used in many fields, such as agriculture [14], medicine [15], and food processing [16], and it is also suitable for real-time detection of different parameters in microbial fermentation process [17,18].

In order to quickly and efficiently screen high-yield gellan gum mutants, ARTP mutagenesis technology and NIRS detection technology were applied in this study. An on-line and rapid NIRS detection model of gellan gum was established, which was used to screen high-yield gellan gum mutants after ARTP mutagenesis. The genetic stability of the screened mutant strains was verified by traditional alcohol precipitation method. Finally, the fermentation conditions of the screened mutant strains were optimized using single-factor experiments and response surface methodology.

## 2. Materials and Methods

### 2.1. Microorganism and Cultivation

The strain used in this study was *S. paucimobilis* ATCC 31461 purchased from American type culture collection (ATCC), and it was maintained on solid medium containing (g/L): beef extract 3, tryptone 5, sodium chloride 15, and agar powder 15. The single colony was cultured in seed medium containing (g/L): yeast powder 1, beef extract 3, tryptone 5, NaCl 5, and sucrose 5. At the later logarithmic stage, 10% inoculation amount was inoculated into fermentation medium (g/L) (yeast powder 0.2, tryptone 2, sucrose 30, KH_2_PO_4_ 1, K_2_HPO_4_ 1.5, and MgSO_4_ 0.6) and cultured for 3 days. The *S. paucimobilis* ATCC 31461 and its mutant strains were cultured at 30 °C in different media.

### 2.2. Growth Curve of Seed Liquid

A ring of activated *S. paucimobilis* ATCC 31461 strain was inoculated in a 250 mL flask containing 50 mL seed culture medium and cultured in a shaking table at 220 r/min. Samples were taken every 4 h and measured by ultraviolet spectrophotometer at 600 nm with culture medium as control. The growth curve of *S. paucimobilis* seed solution was drawn with time as abscissa and absorbance of samples as ordinate.

### 2.3. Determination of Gellan Gum Yield and Fermentation Broth Viscosity

The amount of 10.00 g of fermented broth of the *S. paucimobilis* ATCC 31461 or the mutant strain was diluted with 90 mL distilled water, heated in a 95 °C water bath for 15–20 min, and 200–300 mL of 95% alcohol was added. The solution was left at 4 °C overnight and centrifuged at 4000 rpm for 15 min to precipitate gellan gum [19]. The gellan gum was dried at 60 °C and weighed. The viscosity of fermentation broth was measured at the rotation speed of 0.6 r/min with a viscometer (NDJ-8S, Sdmeik, Qingdao, China).

### 2.4. ARTP Mutagenesis

The solution of the *S. paucimobilis* ATCC 31461 strain was diluted with sterile normal saline until the OD 600 was 0.6–0.8 and mixed with 10% (*v*/*v*) glycerol in a ratio of 1:1. Then, 20 μL was evenly spread on the sterile metal substrate and placed in the ARTP mutagenic device (ARTP-IIS, TMAXTREE, Wuxi, China). The power was set to 100 W and the gas volume was set to 10 SLM [20]. The treatment time was 0–120 s. After the treatment, the bacterial liquid was diluted into different gradients and coated on solid medium, and cultured in an incubator at 30 °C for 4–6 days. The fatality rate under different treatment time was calculated to determine the optimal treatment time.

### 2.5. Collection and Model Building of NIRS

Samples of the *S. paucimobilis* ATCC 31461 or the mutant strains were taken at intervals of 2/4 h within the fermentation time range of 6–72 h. Near-Infrared Spectrometer (Antaris II, Thermo, Waltham, MA, USA) was used to scan different sites of fermentation broth by fiber optic probe, and the background was corrected by instrument internal reference. Eight batches of fermentation were carried out, and spectral information of 78 fermentation samples was collected. The yield of gellan gum was determined by alcohol precipitation method for the establishment of NIRS model.

Different methods were used for pretreatment of NIRS, including standard normal transformation (SNV), multivariate scatter correlation (MSC) and smooth filtering (Savitzky–Golay, S-G), normalization, and derivative processing. The full spectrum (4000–10,000 cm^−1^) of samples was divided into 15 sub-regions, and the synergy interval partial least squares (siPLS) method was used to combine four sub-intervals to establish the NIRS model. The correlation coefficients between model training set and prediction set (r_c_ and r_p_, respectively), root mean square error of the training set (RMSECV), and root mean square error of the prediction set (RMSEP) were used as the measurement standards of the model [21,22,23].

### 2.6. Screening of High-Yield Mutant Strains

After the optimal ARTP treatment, the solution of the *S. paucimobilis* ATCC 31461 strain was evenly coated on the solid medium containing ampicillin (100 mg/mL) and cultured for 4–6 days. Then the large, round, and smooth pale yellow single bacterial colony was cultured on the solid medium for 3–5 days and inoculated into the liquid medium for fermentation. The yield of gellan gum was predicted by constructed NIRS model, and the high-yield mutant strain was selected.

### 2.7. Single-Factor Screening Experiments

The yield of gellan gum and viscosity of fermentation broth produced by the mutant strain 519 were used as evaluation indexes; only one factor was analyzed, and other fermentation conditions were consistent with the original fermentation conditions in a single experiment. The composition of initial medium (*w*/*v*) was as follows: 3% sucrose, 0.2% peptone, 0.1% KH_2_PO_4_, 0.15% K_2_HPO_4_, and 0.06% MgSO_4_ (pH 7.0). After 20 h of seed liquid culture, the seed liquid was inoculated into fermentation broth at 10% and cultured at 30 °C. The factors analyzed in this study included: carbon sources (glucose, sucrose, lactose, maltose, and trehalose); glucose concentrations (1, 2, 3, 4, and 5%) (*w*/*v*); nitrogen sources (yeast powder, peptone, soybean meal, and corn steep liquor dry powder); soybean meal concentrations (0.1, 0.2, 0.3, 0.4, and 0.5%) (*w*/*v*); inoculation amount (4, 6, 8, 10, 12, and 14%); initial pH (6.0, 6.5, 7.0, 7.5, and 8.0); culture time of seed liquid (14, 16, 18, 20, 22, and 24 h); and different proportion of fermentation liquid to bottle volume (5, 10, 15, 20, 25, and 30%).

### 2.8. Response Surface Optimization Experiment

On the basis of the results of single-factor experiments, taking the gellan gum yield of the mutant strain 519 as the response value, the response surface design of three significant factors was carried out using Design Export software as follows: soybean meal concentration (0.3, 0.4, and 0.5%), inoculation amount (4.0, 6.0, and 8.0%), and initial pH (7.0, 7.5, and 8.0). The obtained optimal fermentation conditions after response surface optimization were verified by three biological experiments.

### 2.9. Statistical Analysis

Statistical analysis was conducted using a SPSS 16.0 software (IBM, Armonk, NY, USA) with a significance level of *p* < 0.05. All graphs were drawn with the Origin Pro software (Origin Lab Corporation, Northampton, MA, USA).

## 3. Results

### 3.1. Lethality Rate of S. paucimobilis by ARTP Mutagenesis

**Figure 1:**
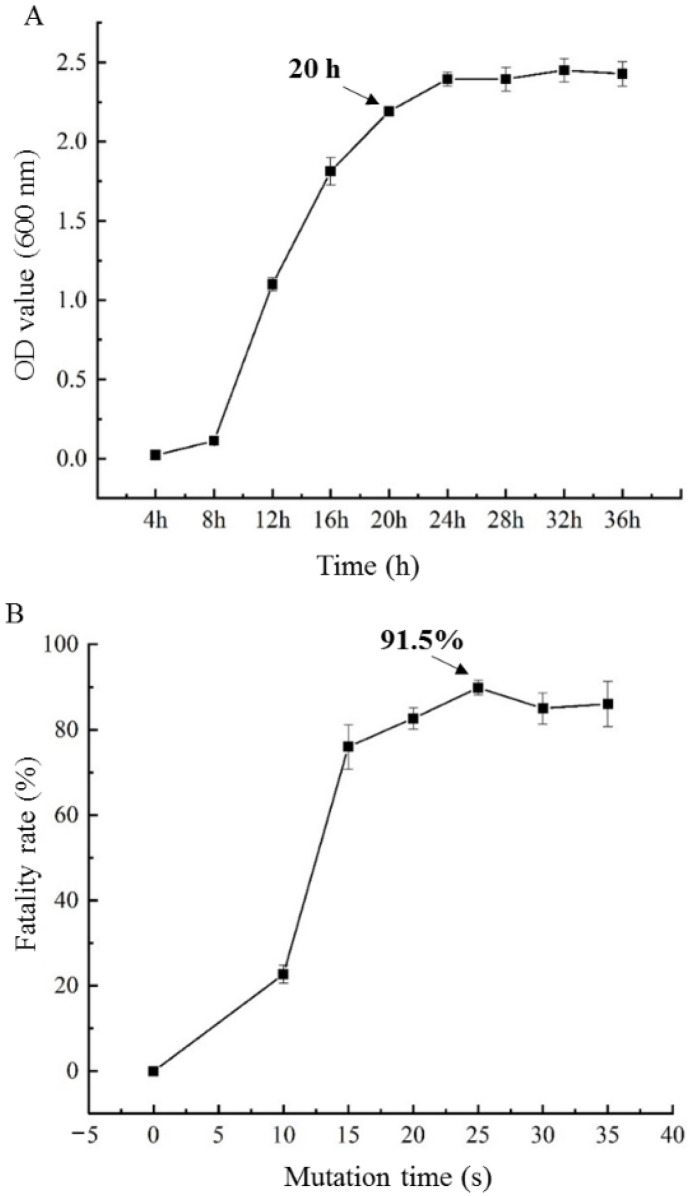
The growth curve (**A**) and the mortality rate treated by atmospheric and room temperature plasma (ARTP) (**B**) of *S. paucimobilis* ATCC 31461. The arrow in (**A**) indicates the optimal culture time point for ARTP mutation.

The growth curve of *S. paucimobilis* ATCC 31461 reflects its growth state (Figure 1A), which is conducive to determining the best growth period of the original strain induced by ARTP treatment. The initial stage of culture (0–8 h) was the growth stagnation stage, then it entered the logarithmic growth stage (8–24 h) and the stable stage after 24 h. Because strains had high vitality in the late logarithmic growth period, the seed solution after 20 h growth was selected for mutagenesis.

The seed solution of *S. paucimobilis* ATCC 31461 was mutagenized for 5, 10, 15, 20, 25, 30, and 35 s, and the mortality of the strains treated by ARTP was calculated with the untreated strains as the control. As shown in Figure 1B, the mortality increased with the extension of ARTP mutagenesis time within 0–25 s, and the highest mortality rate was 91.5% when ARTP mutagenesis time was 25 s. According to the literature reports [13], when the mortality rate of ARTP was in the range of 90–95%, the positive mutation rate was relatively higher, which was conducive to screening out mutants with excellent traits. If the mortality rate was higher than 95%, the negative mutation rate increased and the positive mutation rate decreased [24,25]. Therefore, 25 s was selected as the best ARTP mutation time to screen high-yield gellan gum mutants.

### 3.2. The Construction of NIRS Model for the On-Line Detection of Gellan Gum Yield

Various mutants were induced by ARTP mutation. To screen the strains with stable heredity and higher yield of gellan gum, it was necessary to screen many mutants, and the workload was very heavy. Moreover, the traditional alcohol precipitation method requires a large quantity of chemical reagents to measure gellan gum yield in the screening process, which was costly and not conducive to environmental protection. Therefore, it is very necessary to establish a Near-Infrared on-line detection model for the yield of gellan gum, which can be used to screen high-yield gellan gum mutants efficiently.

#### 3.2.1. Chemical Determination and NIRS Acquisition of Gellan Gum Yield

**Figure 2:**
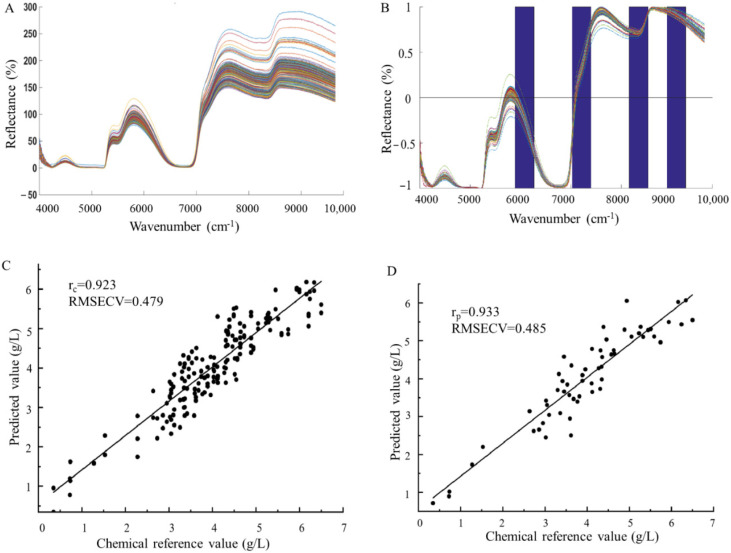
Near Infrared Spectroscopy (NIRS) acquisition and model construction of gellan gum yield. (**A**) Original spectrogram; (**B**) Best subinterval; (**C**) Training set; and (**D**) Prediction set.

The original strain (*S. paucimobilis* ATCC 31461) and its mutant strains were selected for liquid fermentation, and NIRS were collected at different time points. Three different sites of each sample were scanned, and 234 spectra were collected from 78 samples (Figure 2A). Simultaneously, the gellan gum yield in fermentation broth was measured according to the traditional alcohol precipitation method, and the gellan gum yields of 78 samples were measured (Table 1), which were used to establish the NIRS model.

#### 3.2.2. Establishment and Verification of Optimal NIRS Model by siPLS Regression

The original NIRS is easily interfered by environmental background, baseline drift, and other factors. In order to improve the accuracy of modeling, six preprocessing methods were used in this study to preprocess the spectrum, including SNV, MSC, normalization, S-G, D1, and D2 (Figure S1). In order to improve the correlation between the target content in the sample and the spectral information, the Fourier NIRS range of 4000~10,000 cm^−1^ was divided into 15 sub-intervals, and the gellan gum yield and the spectral information of four intervals were modeled by siPLS. As shown in Figure 2B, the best joint subintervals for gellan gum yield modeling were 6008–6405 cm^−1^, 7210–7607 cm^−1^, 8412–8809 cm^−1^, and 9210–9603 cm^−1^.

The correlation coefficient and root mean square error were used to select the optimal model established by different pretreatment methods for the prediction of gellan gum yield. The results are shown in Table 2 and Figure 2C,D. The best pretreatment method was normalization, the best principal component number was 8, the correlation coefficients of training set and prediction set were 0.9230 and 0.9328, respectively, and the root mean square errors of the calibration and prediction sets were 0.4790 and 0.4850, respectively, which indicates that the NIRS technology can on-line detect the yield of gellan gum in the fermentation.

### 3.3. Screening of High-Yield Gellan-Gum-Producing Mutants Using the NIRS Model

**Figure 3:**
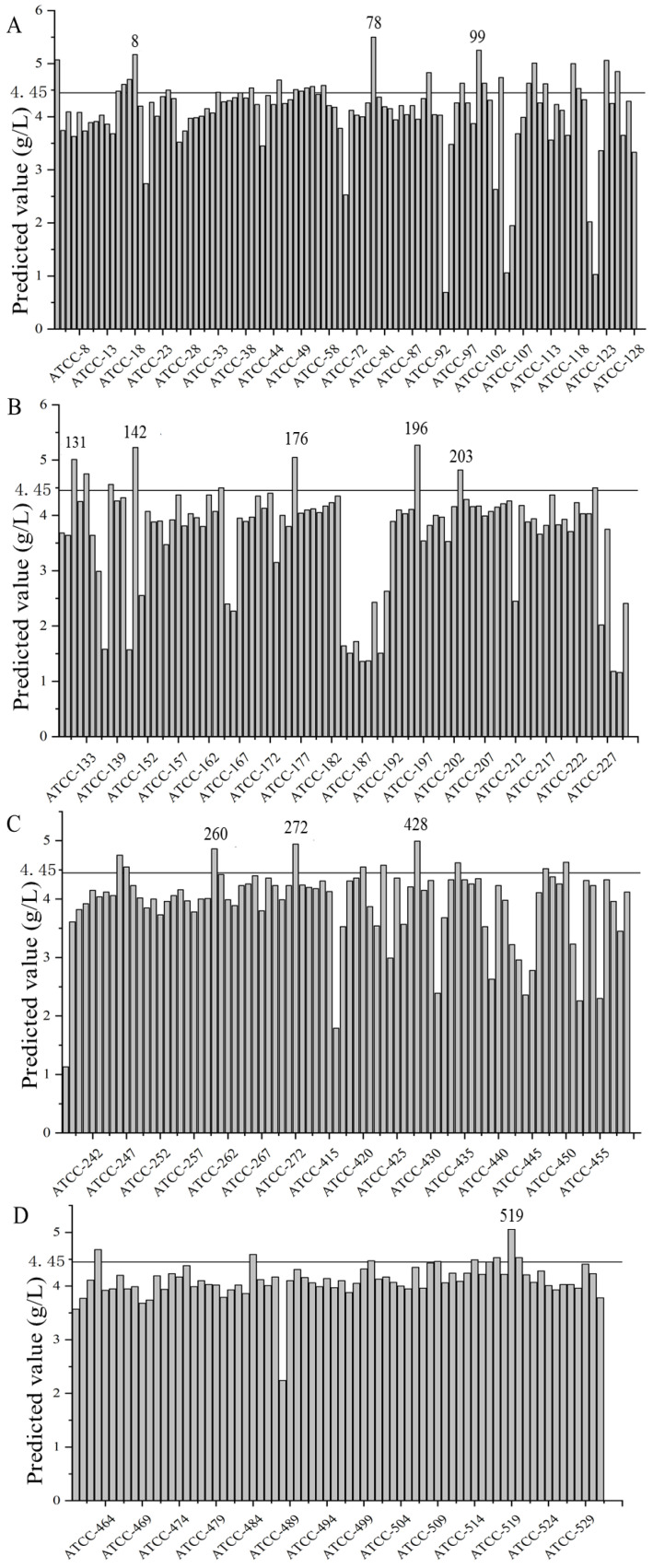
The predicted yield of gellan gum from mutant strains induced by ARTP. (**A**) the mutant strain 1–128; (**B**) the mutant strain 129–230; (**C**) the mutant strain 238–276, 415–459; (**D**) the mutant strain 460–531.

The solution of *S. paucimobilis* ATCC 31461 strain treated by ARTP for 25 s was diluted to different concentrations, coated on the solid medium containing ampicillin, and cultured at 30 °C for 4–6 days (Figure S2). The light yellow, larger, and smooth colonies were inoculated in a shake flask. Using the NIRS model established, the gellan gum yield in fermentation broth was quickly predicted. The predicted yields of gellan gum from approximately 600 mutant strains are shown in Figure 3. Among them, compared with the original strain (yield: 4.45 g/L), the gellan gum yield of 17 mutant strains significantly increased, marked as 3, 18, 78, 95, 99, 110, 117, 123, 131, 142, 176, 196, 203, 260, 272, 428, and 519. Meanwhile, approximately 30 mutant strains had a significantly decreased yield of gellan gum.

### 3.4. Genetic Stability of the High-Yield Gellan Gum Mutants

**Figure 4:**
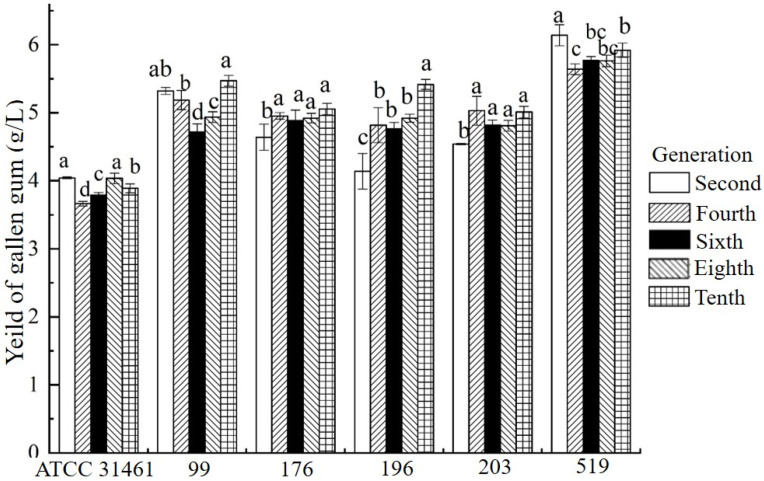
Genetic stability of the high-yield gellan gum mutants within ten generations. Different letters represent a significant difference between the two in a strain (*p* < 0.05).

The 17 mutant strains obtained above were continuously passaged for ten generations, and the yields of gellan gum were measured by the alcohol precipitation method to analyze the genetic stability of the mutant strains. The gellan gum yields of mutant strain 99, 176, 196, 203, and 519 within ten generations were relatively stable (Figure 4). Remarkably, the gellan gum yield of mutant strain 519 was up to 6.32 g/L, which increased by 42.0%, compared with the original strain. Then, the fermentation conditions of mutant strain 519 were optimized. In addition, the gellan gum yield of other mutant strains decreased obviously during passage, and some of them were even lower than that of the original strains, which may be due to the reversion mutation.

### 3.5. Optimizing Fermentation Conditions of Mutant Strain 519 by Single-Factor Experiments

#### 3.5.1. Different Carbon Sources and Optimum Concentration

**Figure 5:**
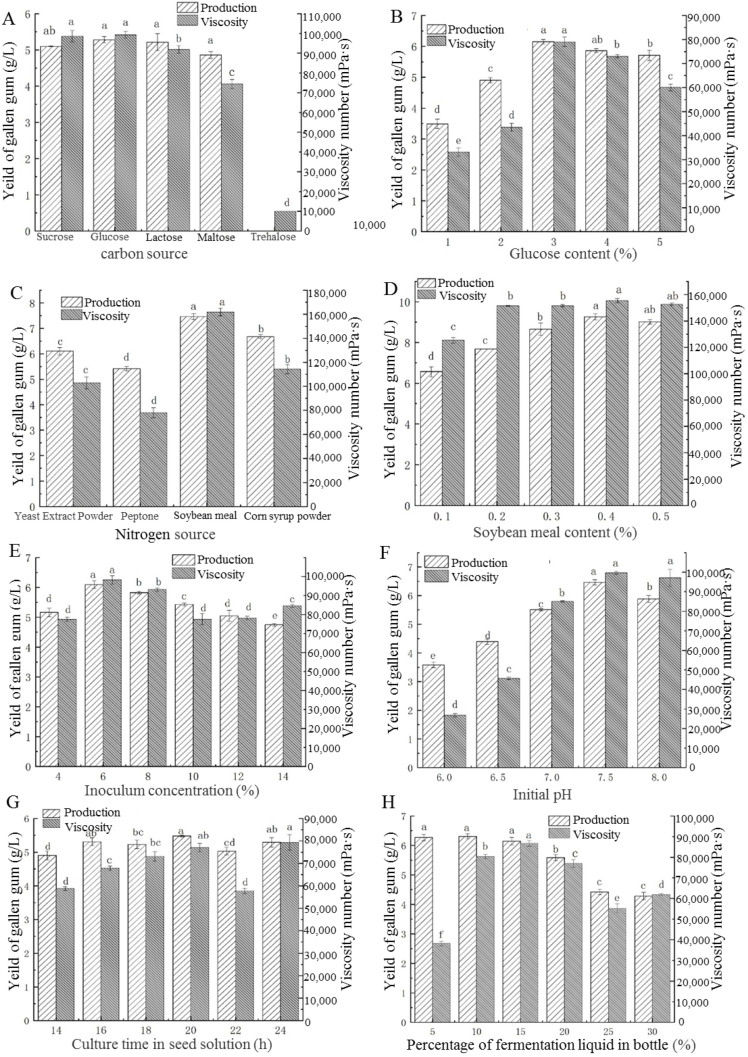
Optimization of fermentation conditions of mutant strain 519 by single-factor experiments. (**A**) Carbon sources; (**B**) Glucose content; (**C**) Nitrogen sources; (**D**) Soybean meal content; (**E**) Inoculum concentration; (**F**) Initial pH; (**G**) Culture time in seed solution; and (**H**) Percentage of fermentation liquid in bottle. Different letters represent a significant difference between the two in a strain (*p* < 0.05).

Fermentation conditions are important factors affecting the synthesis of gellan gum. As shown in Figure 5A, different carbon sources have a great influence on the yield of gellan gum and the viscosity of fermentation broth. When glucose was used as carbon source, the yield of gellan gum and the viscosity of fermentation broth were the highest. In addition, when sucrose, lactose, and maltose were each used as carbon sources, the yield of gellan gum was relatively higher. However, when trehalose was used as carbon source, hardly any gellan gum was produced. Considering the yield of gellan gum, the viscosity of fermentation broth, and lower production cost, glucose was selected as the best carbon source for the fermentation of gellan gum.

Different glucose concentrations also have great influence on the fermentation of gellan gum (Figure 5B). With the increase of glucose concentration, the yield of gellan gum and the viscosity of fermentation broth increased at first and then decreased. When the glucose concentration was 3%, the yield of gellan gum (6.14 g/L) and the viscosity of fermentation broth reached the maximum. Therefore, 3% glucose was the best carbon source concentration for the fermentation of gellan gum.

#### 3.5.2. Different Nitrogen Sources and Optimum Concentration

Figure 5C shows the effects of different nitrogen sources (yeast powder, peptone, soybean meal, and corn steep liquor dry powder) on the yield of gellan gum and the viscosity of fermentation broth. Significantly, when soybean meal was used as nitrogen source, the yield of gellan gum (7.45 g/L) and the viscosity of fermentation broth (160,900 cp) were both at a maximum. Moreover, the cheaper soybean meal, a by-product of soybean oil extraction, is beneficial for reducing the production cost of gellan gum. Therefore, the best nitrogen source for gellan gum fermentation was soybean meal.

As shown in Figure 5D, with the increase of soybean meal concentration (0.1, 0.2, 0.3, 0.4, and 0.5%), the gellan gum yield and the viscosity of fermentation broth showed a trend of increasing. When the soybean meal concentration was 0.4%, the gellan gum yield (9.37 g/L) and the viscosity of fermentation broth reached their highest values. Therefore, the optimum soybean meal concentration of mutant strain 519 for the gellan gum fermentation was 0.4%.

#### 3.5.3. Different Inoculation Amounts

Different inoculation amounts (4, 6, 8, 10, 12, and 14%) affected the synthesis of gellan gum (Figure 5E). When the inoculation amount was 6%, the yield of gellan gum and the viscosity of fermentation broth reached the maximum. When the inoculation amount further increased, the yield of gellan gum decreased. This may be due to the fact that the higher amount of inoculation caused the bacteria to propagate too quickly and had higher nutrient consumption in the early stage, which led to the lack of nutrition in the later stage and restricted the synthesis of gellan gum. Therefore, the optimum inoculation amount for the fermentation of gellan gum was 6%.

#### 3.5.4. Different Initial pH Values

Different initial pH values of the culture medium significantly affected the synthesis of gellan gum (Figure 5F). With the increase of the initial pH value (6.0, 6.5, 7.0, 7.5, and 8.0), the yield of gellan gum and the viscosity of fermentation broth increased at first and then decreased. When the initial pH value of the culture medium was 7.5, the yield of gellan gum and the viscosity of fermentation broth reached the maximum, so the optimal initial pH value of the culture medium for the fermentation of gellan gum was 7.5.

#### 3.5.5. Different Culture Times in Seed Solution

As shown in Figure 5G, the culture time of seed solution inoculated into the fermentation broth had little effect on the yield of gellan gum but had great effect on the viscosity of gellan gum. When the seed solution cultured for 20 h was inoculated, the yield of gellan gum and the viscosity of fermentation broth reached the maximum. Therefore, the seed liquid cultured for 20 h was selected for the fermentation of gellan gum.

#### 3.5.6. Different Proportions of Fermentation Liquid to Bottle Volume

The proportion of fermentation liquid to bottle volume affects the synthesis of gellan gum (Figure 5H) because *S. paucimobilis* is an aerobic bacterium. When the proportion of fermentation liquid to bottle volume was in the range of 5–15%, the yield of gellan gum reached a similar highest level, and the viscosity of fermentation broth increased with the increase of fermentation liquid. When the liquid content was 20%, the yield of gellan gum and the viscosity of fermentation broth decreased slightly. When the proportion of fermentation liquid to bottle volume increased to the range of 25–30%, the yield of gellan gum and the viscosity of fermentation broth were the lowest, which may be due to the decrease of dissolved oxygen affecting the growth of bacteria and the synthesis of gellan gum. Considering the production cost and capacity of gellan gum, the optimum proportion of fermentation liquid to bottle volume was 20%.

### 3.6. Optimizing Fermentation Conditions of Mutant 519 by Response Surface Methodology

#### 3.6.1. Establishment of the Response Surface Model

On the basis of the above results of single-factor experiments, three factors that significantly affected on the yield of gellan gum were obtained: soybean meal content, inoculation amount, and initial pH value. The Design Export software was used to design the response surface optimization experiment (Table 3), and the quadratic multinomial regression equation among soybean meal concentration, inoculation amount, and initial pH value was obtained:

*Y* = −48.20299 + 23.26765 × *A* + 0.148629 × *B* + 13.82214 × *C* + 0.068750 × *AB* +

0.525000 × *AC* + 0.066250 × *BC* − 30.10164 × *A*^2^ − 0.054041 × *B*^2^ − 0.963649 × *C*^2^

**Figure 6:**
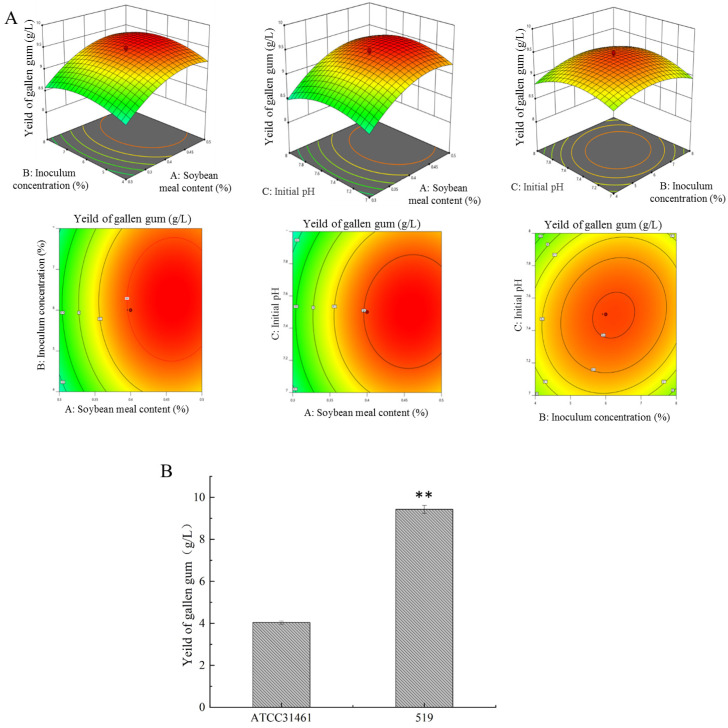
Optimization of fermentation conditions of mutant by Response Surface Methodology. (**A**) The effect of the interaction of fermentation conditions on the yield of gellan gum; (**B**) The experimental verification of the optimal fermentation conditions and yield. “**” represents a significant difference *(p* < 0.01).

The variance analysis of the results was carried out by using this equation, and the results are shown in Table 4. The *p* < 0.0001 of the model and the error of mismatch *p* = 0.2400 (*p* > 0.05) indicated that the established model fitted well with the test data. The correlation coefficient was 98.49%, and the adjusted correlation coefficient was 97.12%, which indicates that the prediction result of this model was influenced mainly by experimental factors. Therefore, this model can be used for the analysis and prediction of gellan gum yield. According to the significance analysis, the order of influence of various factors on gellan gum yield was soybean meal concentration (*A*) > inoculation amount (*B*) > initial pH value (*C*). As shown in Figure 6A, the interaction of soybean meal content, inoculum size, and initial pH value had a great influence on gellan gum yield, and the interaction between inoculum size and initial pH value was significant (*p* < 0.05). The order of the influence of interaction on gellan gum yield was *BC* > *AC* > *AB*.

#### 3.6.2. Prediction and Verification of the Optimal Fermentation Conditions

According to the prediction of response surface model, the optimal fermentation conditions were as follows: the addition amount of soybean meal (A) was 0.459%, the inoculation amount (B) was 6.275%, and the initial pH (C) was 7.513. Under these fermentation conditions, the predicted yield of gellan gum was 9.524 g/L. To verify these results, three independent experiments were carried out under the conditions of 0.46% soybean meal addition, 0.63% inoculation, and initial pH of 7.5 (fine-tuned for the convenience of experiments). As shown in Figure 6B, the average gellan gum yield was 9.427 g/L, which was close to the predicted yield of 9.524 g/L, indicating that the response surface optimization model was reliable. Compared with the original strain ATCC 31461, the gellan gum yield of mutant strain 519 increased by 133.5% (*p*-value < 0.01) under the optimal fermentation conditions.

## 4. Discussion

In recent years, gellan gum has been widely used in food, pharmaceuticals, biomedicine, microbiology, plant tissue culture, and many more applications due to its excellent gel properties, stability, adaptability, etc. [5,6,7]. However, low production yield, high downstream extraction cost, and abundant market demand have made gellan gum a high-priced material [26]. Genetic modification is still an important way to increase yield. West [27] used 1% EMS to treat *Pseudomonas* sp. ATCC 31461 and obtained a mutant strain with elevated production of gellan gum. Arockiasamy et al. [28] analyzed the nonionic surfactants, including Tween 80, Tween 40, and Triton X-100, to improve gellan gum production of *S. paucimobilis*, and obtained the maximum yield (10.44 g/L) with Triton X-100 at 0.75 g/L. Li et al. [7] constructed a carotenoid- and poly-β-hydroxybutyrate-free mutant strain of *Sphingomonas elodea* ATCC 31461 by knocking out the phytoene desaturase gene (crtI) and phaC gene, combining UV irradiation and EMS mutagenesis treatment to elevate gellan gum production. However, similar works in the literature to elevate gellan gum production were poor. Recently, as a new biological mutagenesis technology, ARTP has been successfully applied in microbial mutagenesis breeding due to its advantages of simple operation, mild conditions, high positive mutagenesis rate, and genetic stability [9,11,29]. In this study, the fatality rate of *S. paucimobilis* ATCC 31461 reached 91.5% after ARTP mutagenesis for 25 s. On the basis of the high efficiency of mutagenesis when the fatality rate was approximately 90–95% [13], 25 s was selected as the best ARTP mutation time for the screening high-yield mutant strains. Moreover, this ARTP treatment produced a variety of mutant types based on gellan gum yield and achieved good mutagenic effect. Similarly, Liu et al. [29] selected 15 s of ARTP treatment with a 93.84% mortality rate of *B. coagulans* WT-03 as the optimum treatment time and obtained a mutant artp-aleBC15 with improved tolerance after 15 s of ARTP mutation and 40 days of ALE culture.

ARTP treatment is often used in combination with other methods to improve mutagenesis efficiency. Liu et al. [29] combined ARTP treatment with ALE to improve the probiotic performance of *B. coagulans* WT-03. Gu et al. [30] proved that ARTP/EMS-combined mutagenesis efficiently improved production of raw-starch-degrading enzymes in *Penicillium oxalicum*. In the process of ARTP mutagenesis, the high concentration of neutral active ions directly penetrates the cell membrane into the nucleus and causes gene damage, thus causing various types of mutations [9]. Although ARTP has high mutagenesis efficiency, it is a random mutagenesis method. Therefore, only by screening a large number of mutant strains can strains with good performance be detected and screened. In this study, an efficient mutagenesis and rapid screening method of high-yield gellan gum mutants by combining ARTP mutation and NIRS detection technology was proposed for the first time. By using the optimal normalization pretreatment method and the siPLS regression method, the NIRS model for on-line predicting the yield of gellan gum in fermentation broth was established, which can replace the traditional alcohol precipitation method to determine the yield of gellan gum. Based on this, the screening efficiency was greatly improved, and the mutant strain library could be expanded to facilitate the screening of more mutant strains with beneficial traits. On the other hand, the alcohol precipitation method, commonly used to determine the yield of gellan gum, requires the use of alcohol or isopropyl alcohol 2–3 times that of the fermentation liquid and 1–2 days to determine the yield of gellan gum, which is time-consuming, laborious, and not conducive to environmental protection. The NIRS model established in this study can directly predict the yield of gellan gum in fermentation liquid, which saves time and labor and is environmentally friendly. In addition, the NIRS model can also be used to on-line predict the yield of gellan gum during fermentation in the industrial production process.

The gellan gum yield of approximately 600 mutant strains was predicted on the basis of the established ARTP high-efficiency mutation and NIRS model. The yield of gellan gum did not change significantly in most of mutant strains. Significantly, only 17 mutant strains had a higher yield, while approximately 30 mutant strains had a decreased yield of gellan gum. It also proved that ARTP mutagenesis is relatively random, and a large number of mutant libraries need to be screened to detect the strains with high-yield gellan gum. Further, 17 mutant strains were passed for ten generations and gellan gum content was measured by alcohol precipitation method. Only five mutant strains had stable gellan gum yield within 10 generations, and the other strains had no significant yield increase. Similar phenomena were also found in the report of Xiang et al. [31] due to the reverse mutation.

Among the high yield and stable mutant strains, the mutant strain 519 showed the largest yield improvement. Single-factor and response surface experiments were used to optimize the fermentation conditions of mutant strain 519, and the yield of gellan gum increased by 133.5%. Different fermentation conditions, including carbon sources, nitrogen sources and their concentrations, inoculation amounts, initial pH, culture time in seed solution, and proportion of fermentation liquid to bottle volume, had a great impact on the gellan gum yield of mutant strain 519 (Figure 5). The most important factors affecting the gellan gum yield of mutant 519 were as follows: soybean meal concentration (A) > inoculation amount (B) > initial pH value (C). In addition, the interaction of soybean meal content, inoculum size, and initial pH value had a great influence on gellan gum yield, and the interaction between inoculum size and initial pH value was significant (*p* < 0.05). Therefore, the medium components and their fermentation conditions significantly affected the yield of gellan gum, which was similar to previous reports [32,33,34]. Zhang et al. [34] improved gellan gum production by optimization of culture medium compositions. Huang et al. [32] revealed that corn steep liquor (CSL), the addition of Triton X-100 surfactant and inorganic nitrogen sources improved the yield of gellan gum. Under the optimal fermentation conditions, the gellan gum yield of mutant strain 519 increased by 133.5%, which laid the foundation for the industrial fermentation of the mutant strain 519.

## 5. Conclusions

In order to improve the yield of gellan gum, ARTP mutagenesis was used to mutate the original strain (*S. paucimobilis* ATCC 31461). When ARTP mutation time was 25 s, the mortality of the strain was 91.5%, which was suitable for screening high-yield mutant strains. On the basis of the traditional chemical determination method and NIRS acquisition, a NIRS model was established by siPLS method for on-line detection of gellan gum yield. Firstly, a large number of mutants were obtained by high-efficiency mutagenesis of ARTP, and then the yield of gellan gum in the fermentation was on-line detected by the NIRS model, and five mutants with stable heredity within ten generations were selected. Notably, the yield of mutant strain 519 increased the most. Finally, the fermentation conditions of mutant strain 519 were optimized by single-factor and response surface methodology. Under the optimal fermentation conditions, the yield of mutant strain 519 was 9.427 g/L, which increased by 133.5% compared with the original strain. Therefore, an efficient method for screening high-yield gellan gum mutants was proposed, which can also be used for screening other high-yield polysaccharide mutants.

## Figures and Tables

**Table 1:** Gellan gum yield of *S. paucimobilis* ATCC 31461 and its mutant strains at different fermentation time determined by physical and chemical methods.

Time (h)	Yield (g/L)	Time (h)	Yield (g/L)	Time (h)	Yield (g/L)	Time (h)	Yield (g/L)
6	0.35	36	3.68	56	4.28	72	4.94
8	0.74	36	3.63	60	3.82	72	4.89
8	0.73	36	4.89	60	3.45	72	5.05
12	1.28	40	3.33	60	4.35	72	6.16
12	1.53	42	3.96	64	3.10	72	6.24
12	2.73	44	3.05	68	3.02	72	6.21
16	2.28	44	3.60	72	3.62	72	5.59
16	3.30	44	3.75	72	3.12	72	5.74
20	2.86	46	4.52	72	4.55	72	5.51
20	3.46	48	3.77	72	4.32	72	5.29
24	3.35	48	3.05	72	4.64	72	5.27
24	2.64	48	3.58	72	4.46	72	5.33
24	4.13	48	3.53	72	4.65	72	5.23
28	3.03	48	4.58	72	4.36	72	6.00
28	4.03	48	3.89	72	4.49	72	6.34
28	3.89	52	4.04	72	4.12	72	5.45
30	3.37	52	4.64	72	4.31	72	6.50
32	3.26	52	4.11	72	4.35	72	5.94
32	4.31	54	3.36	72	4.73		
36	2.95	56	3.42	72	4.39		

**Table 2:** Optimum condition for establishing NIRS models of gellan gum content in the liquid fermentation process after different mathematical pretreatments.

Pretreatment Method	LV	Joint Sections	Calibration Set		Prediction Set	
			r_c_	RMSECV	r_p_	RMSEP
No pretreatment	8	6, 9, 12, 14	0.9230	0.4790	0.9268	0.5250
SNV	8	6, 9, 12, 14	0.9230	0.4790	0.9326	0.4850
MSC	8	6, 9, 12, 14	0.9230	0.4790	0.9327	0.4850
Normalization	8	6, 9, 12, 14	0.9230	0.4790	0.9328	0.4850
S-G	8	6, 9, 11, 14	0.9207	0.4850	0.9133	0.5670
D1	9	6, 8, 10, 14	0.8982	0.5490	0.9087	0.5710
D2	7	4, 9, 13, 14	0.7485	0.8661	0.7827	0.9280

Noting: r_c_ represents the correlation coefficient of the training set; r_p_ represents the correlation coefficient of the prediction set.

**Table 3:** Design and results of response surface experiment.

Number	*A*	*B*	*C*	Yield (g/L)	
	Soybean Meal Concentration (%)	Inoculation Amount (%)	pH	Actual Value	Predicted Value
1	0.30	4.00	7.00	8.38	8.37
2	0.50	4.00	7.00	9.05	9.00
3	0.30	8.00	7.00	8.30	8.31
4	0.50	8.00	7.00	9.04	8.99
5	0.30	4.00	8.00	8.09	8.16
6	0.50	4.00	8.00	8.88	8.90
7	0.30	8.00	8.00	8.29	8.37
8	0.50	8.00	8.00	9.12	9.16
9	0.23	6.00	7.50	8.05	7.97
10	0.57	6.00	7.50	9.12	9.16
11	0.40	2.60	7.50	8.73	8.72
12	0.40	9.40	7.50	8.92	8.89
13	0.40	6.00	6.70	8.69	8.76
14	0.40	6.00	8.30	8.82	8.71
15	0.40	6.00	7.50	9.48	9.42
16	0.40	6.00	7.50	9.51	9.42
17	0.40	6.00	7.50	9.35	9.42
18	0.40	6.00	7.50	9.37	9.42
19	0.40	6.00	7.50	9.37	9.42
20	0.40	6.00	7.50	9.41	9.42

**Table 4:** Results of regression equation variance analysis.

Source	Sum of Squares	Df	Variance	*F*-Value	*p*-Value
Model	4.1500	9	0.4600	72.26	<0.0001
A	1.7100	1	1.7100	267.68	<0.0001
B	0.0328	1	0.0300	5.14	0.0467
C	0.0022	1	0.0022	0.34	0.5744
AB	0.0015	1	0.0015	0.24	0.6368
AC	0.0055	1	0.0055	0.86	0.3745
BC	0.0351	1	0.0351	5.50	0.0409
A^2^	1.3100	1	1.3100	204.66	<0.0001
B^2^	0.6734	1	0.6700	105.54	<0.0001
C^2^	0.8364	1	0.8400	131.09	<0.0001
Residual	0.0638	10	0.0064		
Lack of fit	0.0423	5	0.0085	1.96	0.2400
Pure Error	0.0216	5	0.0043		
Total	4.2100	19			
R^2^	R^2^ = 98.49%				
Adjusted R^2^	R^2^ = 97.12%				

## Data Availability

Data is contained within the article or Supplementary Materials.

## Supplementary Materials

The following supporting information can be downloaded at: https://www.mdpi.com/article/10.3390/foods11244078/s1, Figure S1. The NIRS spectra after different pretreatment methods, including standard normal transformation, SNV (A), multivariate scatter correlation, MSC (B), smooth filtering, Savitzky–Golay, S-G (C), Normalization (D), derivative processing, D1 (E), and D2 (F). Figure S2. The growth state of the mutant strains on solid medium.
