# Supplementary figures and images for: Rapid Screening of High-Yield Gellan Gum Mutants of Sphingomonas paucimobilis ATCC 31461 by Combining Atmospheric and Room Temperature Plasma Mutation with Near-Infrared Spectroscopy Monitoring
Source: Foods. 2022 Dec 16;11(24):4078. doi: 10.3390/foods11244078 (PMC9777525; doi:10.3390/foods11244078)

**Figure S1**

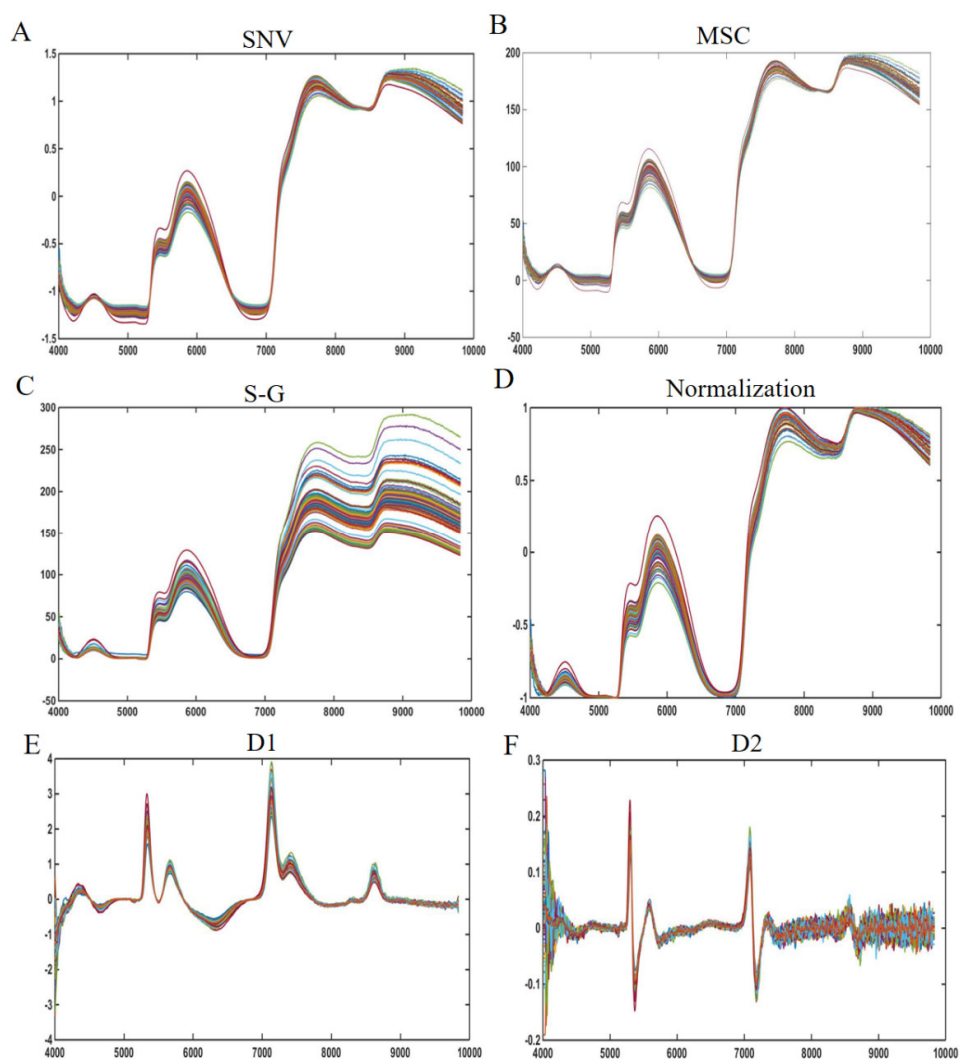

**Figure S2.**

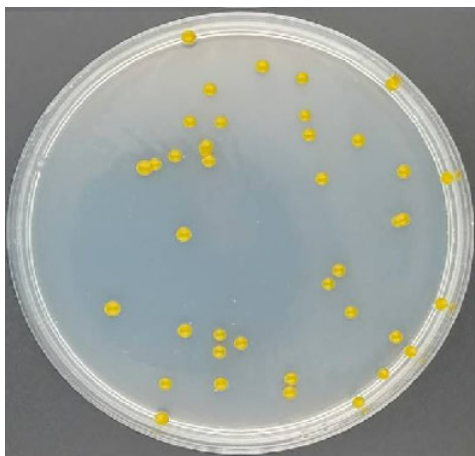

Supplement: Supplementary file 1 [file foods-11-04078-s001.zip › foods-2013717-supplementary-done.pdf]
